# A new feed additive composed of urea and soluble carbohydrate coated with wax for controlled release in ruminal fluid

**DOI:** 10.1038/s41598-022-08372-0

**Published:** 2022-03-16

**Authors:** Alexandre Guimarães Inácio, Camila Celeste Brandão Ferreira Ítavo, Alexandre Menezes Dias, Gelson dos Santos Difante, Joice Ferreira de Queiroz, Lincoln Carlos Silva de Oliveira, Geraldo Tadeu dos Santos, Luís Carlos Vinhas Ítavo

**Affiliations:** 1 Faculty of Veterinary Medicine and Animal Science, Federal University of Mato Grosso do Sul, 2443 Senador Filinto Muller Ave., Campo Grande, MS 79070-900 Brazil; 2 Chemistry Institute, Federal University of Mato Grosso do Sul, 1555 Senador Filinto Muller Ave., Campo Grande, MS 79070-900 Brazil

**Keywords:** Natural product synthesis, Chemical biology

## Abstract

Urea is a compound widely used as a feed additive for ruminants; however, when used profusely, it can lead animals to intoxication. Another factor that affects the effectiveness of urea is the lack of synchronization between the nitrogen and the availability of carbohydrates, necessary for better development of the ruminal microbiota. In order to circumvent these problems and improve the efficiency in urea use, the present study developed two new nutritional additives (F16 and F17) with different carbohydrate sources. One of the products developed (F16) used sugarcane molasses as a carbohydrate source, while the other (F17) used cassava starch. In addition to the carbohydrate source, both products contained the same amounts of urea, sulfur, calcium carbonate and were coated with carnauba wax. The supplements developed and two other commercial products based on extruded urea (UE) and polymer-coated urea (UP) were tested for solubility and cumulative gas production. The wax used in the coating process of the developed products (F16 and F17) proved to be efficient in reducing the solubility of the ingredients used. During chemical composition analysis it was verified that both supplements developed contained protein equivalent above 150% of crude protein. The cumulative gas production showed a higher production related to the product F17 (p < 0.05). Through thermogravimetric analysis, it was found the chemical integrity of the ingredients that make up the supplements developed. Therefore, is possible to reduce the solubility of urea using carnauba wax as a coating material. The formula with cassava starch associated with urea (F17) had a better synchronization during the degradation of its ingredients.

## Introduction

Urea is an organic compound that belongs to the group of non-protein nitrogenous compounds (NPN). In the diet of ruminants, it can be used as an alternative source to true protein, since it is capable of replacing it with the advantage of being less expensive^1^.

Ruminal microorganisms are capable of transforming nitrogen from NPN compounds into protein of high nutritional value. However, if the release of ammonia promoted by NPN exceeds the use capacity by ruminal microbiota, there will be an excretion of this excess with a consequent loss of energy. If the ammonia concentration extrapolates the excretion capacity, the intoxication of the animal may occur^2^.

In order to circumvent these problems and obtain better efficiency in the nitrogen use by ruminal microorganisms, different types of slow-release urea (SRU) such as biuret, starea (extruded urea), urea phosphate, oil-based coatings, urea treated with formaldehyde, and urea coated with polymer were developed^3^. These products decrease the chances of intoxication and can improve bacterial growth, as they are able to maintain a constant availability of nitrogen in the rumen fluid^4^.

The efficiency of products containing SRU is variable. This may occur because the rate of solubilization dependent of the material and method used to coat this urea. For example, a study^5^ found that coating of urea chitosan did not bring significant effect on pH, dry matter and organic matter digestibility, microbial protein synthesis, and amonia concentration in the rumen. On the other hand, some authors^6^ cite that the extrusion of urea with corn, in addition to controlling the release of nitrogen, improves the use of urea due to a better synchronization during the degradation of these ingredients.

The availability of nitrogen (which can be provided by urea) simultaneously with energy (mainly from carbohydrates) are necessary factors for microbial synthesis^7^. Sulfur also influences this effectiveness, as some sulfur amino acids are essential for the development of the ruminal microbiota^2^.

Therefore, the present study aimed to improve the efficiency of using urea by creating two products containing, in addition to urea, a carbohydrate source (sugar cane molasses—F16 or cassava starch—F17), sulfur and calcium carbonate. To get a controlled release, all ingredients were homogenized and coated with carnauba wax, coating material that has desirable characteristics such as hydrophobicity, high melting point, natural origin and is already used in edible food films.

The investigated hypothesis assumes that controlled and synchronized release of the components of these formulations can improve the performance of the rumen microbiota. To evaluate this theory, the developed products and also two commercial SRU products were submitted to solubility analyses, cumulative gas production, chemical, microscopic and thermal analysis.

## Results

### Chemical composition

The chemical analysis of the products developed (F16 and F17), as well as the commercial products extruded urea (UE) and polymer-coated urea (UP) are shown in Table 1. All these compounds were analyzed for dry matter (DM), mineral material (MM), organic matter (OM), ether extract (EE), neutral detergent fiber (NDF) and crude protein (CP). The results of MM and OM are presented as a percentage of DM, while the others are presented as a percentage of the total product.

**Table 1:** Chemical composition of the different nitrogenous products evaluated.

Variables	Nitrogenated products			
	F16	F17	UP	UE
DM (%)	94.41	95.35	99.35	95.03
MM (% of DM)	15.17	13.51	0.14	0.50
OM (% of DM)	84.82	86.48	99.85	99.49
EE (%)	9.01	10.04	4.65	6.87
NDF (%)	7.28	10.79	12.20	2.95
CP (%)	161.42	160.89	265.40	221.26

F16 = urea, sugarcane molasse, sulfur flower and calcium carbonate coated with carnauba wax; F17 = urea, cassava starch, sulfur flower and calcium carbonate; UP = polymer coated urea; UE = extruded urea (starea).

DM: dry matter; MM: mineral matter; OM: organic matter; EE: ether extract; NDF: neutral detergent fiber; CP: crude protein.

### Microscopy

**Figure 1:**
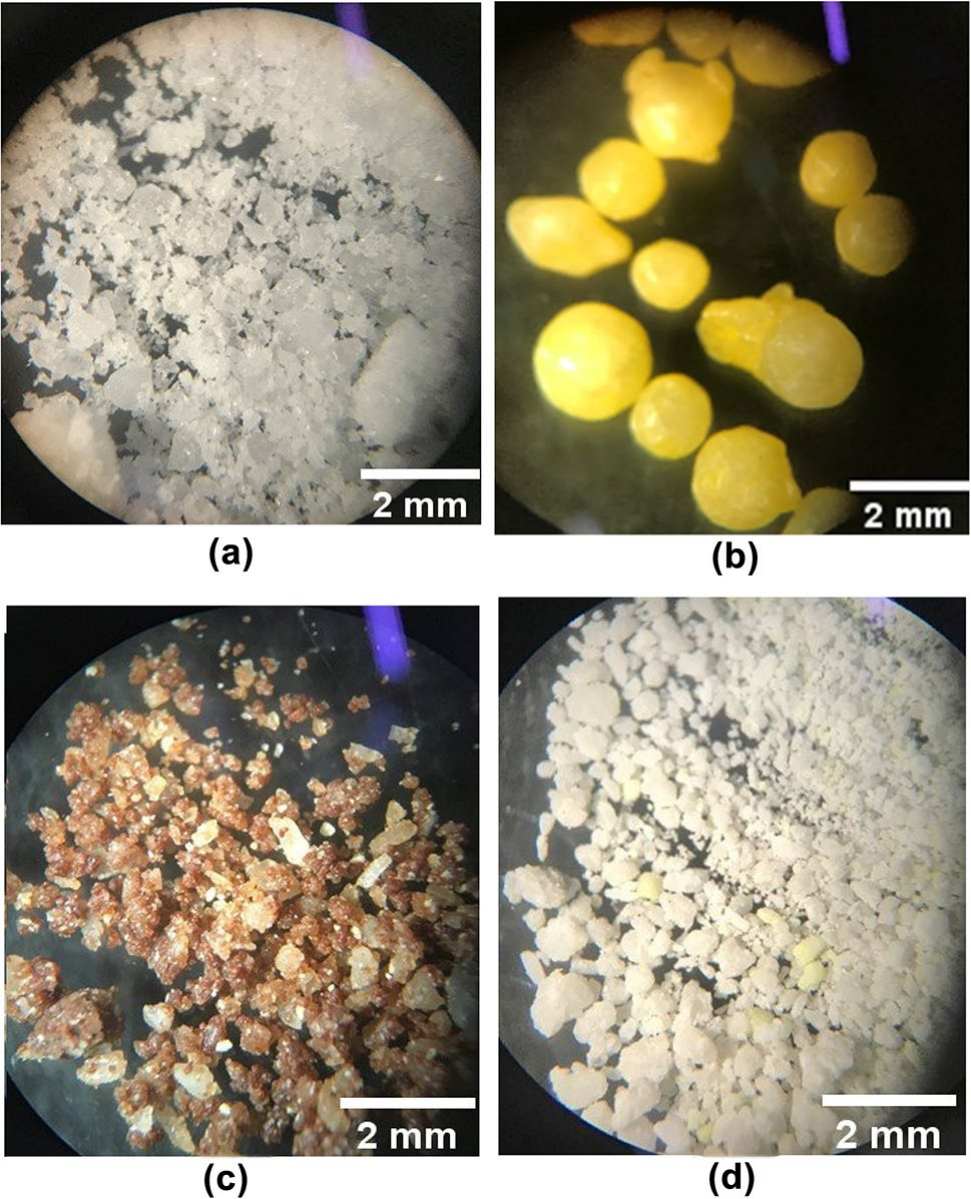
Stereoscopic microscope 40X magnification of nitrogenous products (**a**) Pure urea, (**b**) UP, (**c**) F16, and (**d**) F17.

The granules of pure urea can be seen as the result of the grinding carried out before the analyzes (Fig. 1a). In Fig. 1b, it is possible to observe the intact granules of urea, coated with yellowish polymer (UP). The images in Fig. 1c and d are respectively of products F16 and F17. Granules of brown and yellow color are apparent in Fig. 1c and of white and yellow color in Fig. 1d. These colors were associated with the ingredients that make up these additives.

**Figure 2:**
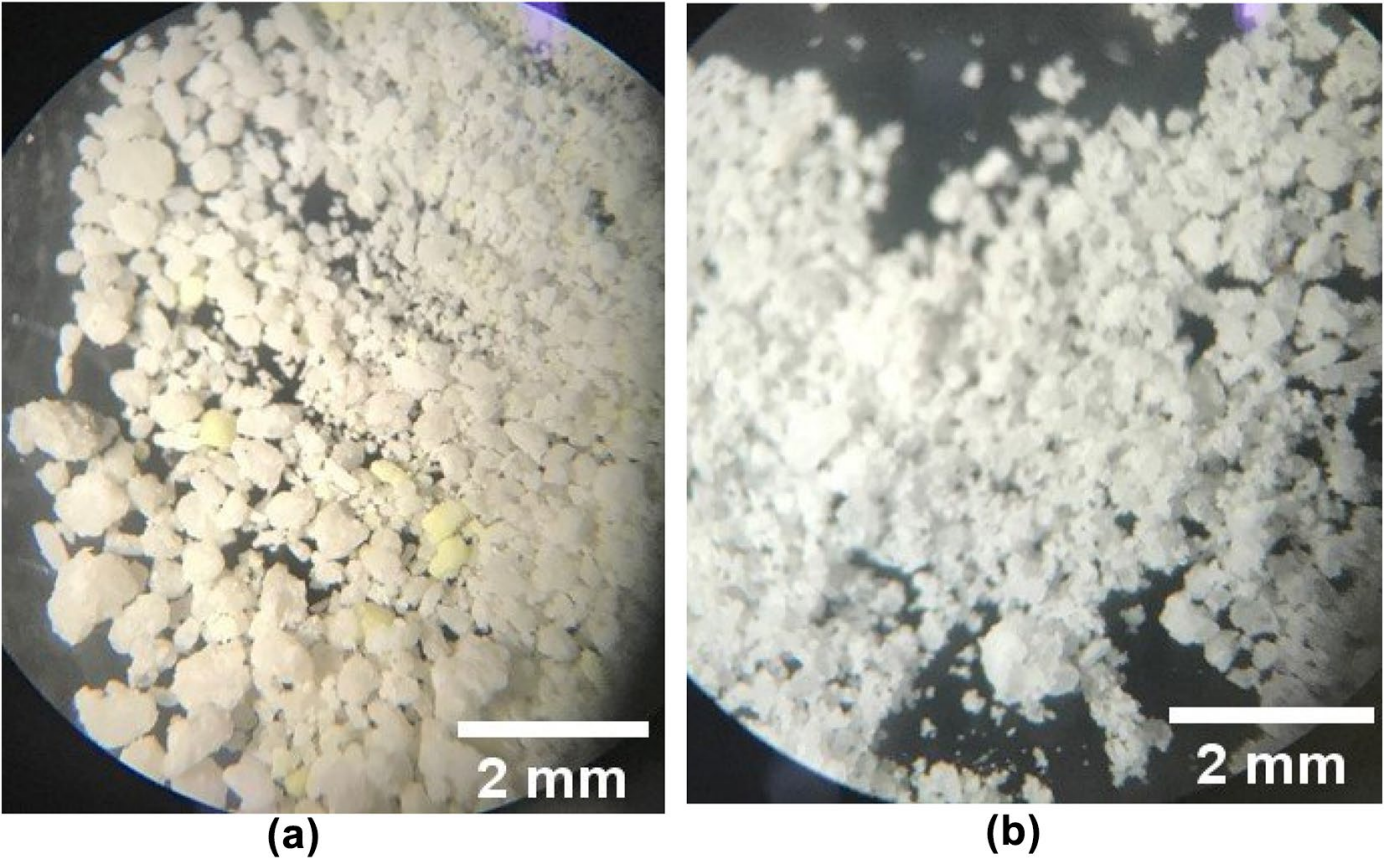
(**a**) F17 complete formulation e (**b**) F17 sulfur-free formulation.

Figure 2a shows the product F17 with its complete formulation, being possible to observe white and yellow granules as in Fig. 1d. Figure 2b shows the image obtained from the product F17 without the addition of sulfur in its formulation, being possible to observe only white granules. In this way, it was possible to verify the influence of sulfur in the appearance of the product and thus discard the lack of homogeneity of the wax during the preparation of the additive.

### Solubilization test

**Figure 3:**
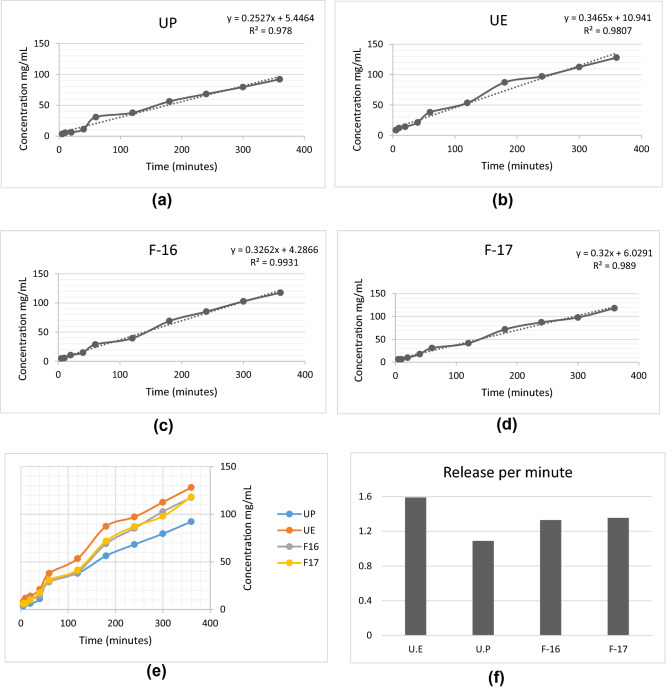
Solubilization test of UP (**a**), UE (**b**), F16 (**c**), F17 (**d**) all tested products (**e**) and Solubilization per minute of all products tested (**f**).

Figures 3a–d contains the results of the solubilization test of the commercial products UP and UE as well as the products F16 and F17. The graph curves in Fig. 3e shows the solubilization of the all tested urea products. In the first hour of analysis, a progressive increase in the concentration of nitrogen was observed in all products, with UP being the least solubilized and UE being the most solubilized. From the first hour there is then a reduction in the speed of solubilization of the products, less for the UE that has a second increase after 120 min. Figure 3f shows the graph with the release rates per minute of the solubilization test. These data corroborate those presented in Fig. 3f with the UE being the product with the highest solubilization, the lowest UP and the products F16 and F17 with intermediate release rates.

### Cumulative gas production

**Figure 4:**
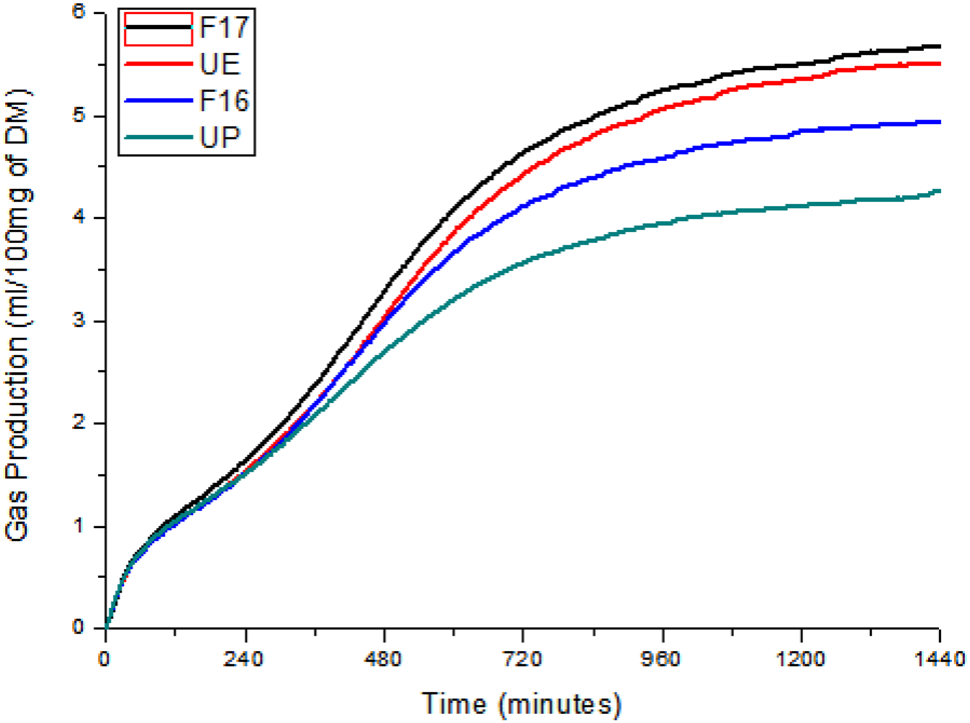
Cumulative gas production of the tested products over 1440 min.

Figure 4 contains the cumulative gas production curves for both commercial products and those developed in this work. The cumulative gas production results exhibit in Fig. 4 shows an increase in production in all samples in the first minutes of analysis. At approximately 120 min of analysis, the F17 product begins to show higher gas production and at around 360 min, it is also possible to see an increase in the curves of the F16 and UE products. At 1440 min, the final analysis time, it was possible to observe a higher gas production related to the product F17, followed by UE, F16 and finally with a lower production the UP.

Table 2 contains the means of the parameters estimated for the production of gas in the different products tested. Gas volume (mL) of the rapidly degradable fraction (A), rate of degradation of the fraction A (B), lag time (C), gas volume (ml) of the slowly degradable fraction (D) and degradation rate of fraction D (E), and Total gas produced (A + D) were evaluated.

**Table 2:** Means of parameters estimated by cumulative gas production in the different nitrogenous products evaluated.

	Nitrogenated products				EPM	p-value
	F16	F17	UP	UE		
A (mL/100 mg DM)	0.26 b	0.26 b	0.38 a	0.22 c	0.015	0.0001
B (%/h)	99.93	99.93	99.93	99.93	0.010	0.9985
C (h)	0.73 c	0.92 b	0.65 d	0.99 a	0.035	0.0001
D (mL/100 mg DM)	4.51 b	5.16 a	4.37 b	5.19 a	0.119	0.0031
E (%/h)	7.85 a	7.87 a	7.86a	7.61 b	0.030	0.0001
Total (A + D) (mL/100 mg DM)	4.77 b	5.42 a	4.75 b	5.41 a	0.109	0.0051
R^2^ (%)	99.85	99.87	99.80	99.90		

Means followed by distinct lower case letters, differ from each other by the Tukey test (P < 0.05).

A = gas volume (ml) of the rapidly degradable fraction; B = degradation rate of fraction A (ml/h); C = lag time (h); D = gas volume (ml) of the slowly degradable fraction; E = degradation rate of fraction D (mL/h).

### Thermogravimetric analysis

**Figure 5:**
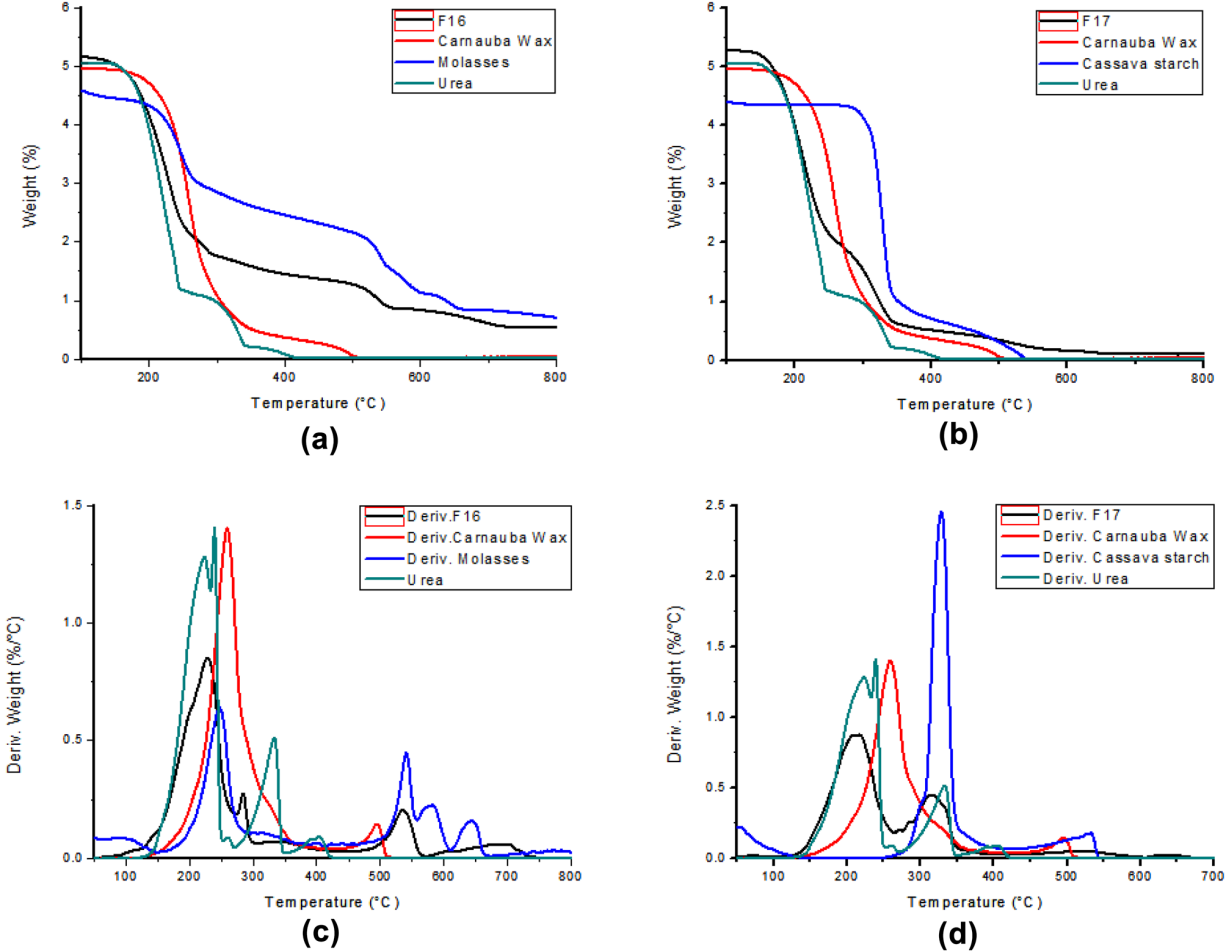
(**a**) Loss of mass of product F16 and its ingredients. (**b**) Loss of mass of product F17 and its ingredients. (**c**) Curves derived first from the loss of mass of product F16 and its ingredients, and (**d**) Curves derived first from loss of mass of the product F17 and its ingredients.

Figure 5 contains the thermogravimetric curves and their derivatives from products F16, F17 and their ingredients. The graphs in Fig. 5a and b show the mass losses that occurred during the analysis, where it is possible to verify the beginning of the event close to 200 °C. The derived curves (Fig. 5c and d) allow more detailed visualization of the thermal events that occurred. It is possible to observe an overlap of the curve of both products (in black) by the curves of the ingredients that compose them (in blue, green and, red).

## Discussion

### Chemical composition

The data from the chemical analysis of the tested products, Table 1, show that the developed feed additives (F16 and F17) reached a value in CP equivalent above the 150% initially proposed in this work. Commercial products (UP and UE) also reached the minimum CP informed by their respective manufacturers.

The amount of DM, MM, OM, and EE found was close among the products developed. This result can be associated with a similar formulation and the identical production methodology of these formulations. The results found for the chemical composition of commercial products corroborate those found in the literature^8,9^.

### Microscopy

Figure 1 contains the images obtained through the stereoscopic microscope of pure urea (Fig. 1a), UP (Fig. 1b) and the products F16 (Fig. 1c) and F17 (Fig. 1d). Figure 1a shows the granules obtained by grinding the urea, it is possible to observe a uniform color, indicating that apparently, there is no presence of impurities. The difference in granule sizes, on the other hand, can be associated with high hygroscopicity of urea, which can cause agglutination of these granules. In Fig. 1b, the urea granules covered with polymer (UP) can be seen. The unevenness of these granules can be associated both with the process of manufacturing urea or with its subsequent coating process with the polymer. A color difference can be observed when comparing Fig. 1c and d, the one showing the product F16 (Fig. 1c) has a darker color. The brown color observed in this product is due to the molasses powder, present in this formulation, which no longer occurs with F17 as it contains cassava starch (white-colored) as a carbohydrate source.

Small yellow granules are also seen in Fig. 1c and d, that could be related to both the sulfur flower and the carnauba wax as both have yellowish color. To clarify the doubt, a sulfur-free F17 formulation was performed (Fig. 2b) and the yellow granules were not observed. Then the yellow granules observed were attributed to the sulfur flower. Although the wax also has a yellow color, it was not observed this image (Fig. 2b).

Failure to observe the wax in the images suggests a good homogeneity in the coating process of the particles, since it would be possible to visualize the wax if there was a poor distribution of this material used in the coating. Homogeneity in the urea coating process is desirable so that the final product has a slow and constant solubilization in the rumen.

### Solubilization test

The solubilization test showed the release of nitrogen in ruminal fluid during 6 h (360 min). Figure 3 shows the concentrations of nitrogen (N) measured in mg/mL. In the first hour of analysis, a rapid increase in N release is observed in all tested products. This fact is due to the rapid hydrolysis that urea undergoes in an aqueous medium. The different liberation rates, also observed among the tested products, reflect the different efficiency of the coatings process.

It can be seen that the UE was the one that released more N (128.04 mg/mL measured at 360 min), that is, this was the least efficient product in reducing urea solubility. On the other hand, UP was the most efficient in preventing the rapid release of nitrogen (92.16 mg/mL measured at 360 min). The products developed in this work had intermediate N release rates (F16 = 117.48 mg/mL and F17 = 118.19 mg/mL measured at 360 min) when compared to commercial products.

Figure 3b shows the results of the same solubilization test, but these data are presented in another way, since they bring the average N release per minute. The results of Fig. 3b corroborate with Fig. 3a showing a higher release of N per minute from the UE (1.58 mg/mL/min), followed by F16 (1.32 mg/mL/min), F17 (1.35 mg/mL/min) and UP (1.08 mg/mL/min). The different release rates observed can be related to the different materials and methods used to coating of these urea sources.

A study^10^ evaluated some materials, in different proportions, used in the urea coating process, and observed varied results in the protection efficiency. These authors reported a relationship between the thickness of the coating and the protection efficiency (the thicker the greater the protection). They also observed that the standardization in the size of the particles, before the coating, increased the protection efficiency. This is because this process allows a better distribution of the coating material.

While the urea of products F16, F17 and UP are protected by coating with hydrophobic material, extruded urea (UE) is obtained using another technique. The UE is a product obtained by extruding a mixture of starch and urea, by means of high temperature and pressure, leading to the gelatinization of the starch. Extrusion causes the incorporation of urea into the starch structure, reducing its solubility^6,11,12^.

The structures created during extrusion depend on the effects induced by heat, pressure and interactions of the feed components chosen for the process^13^. On the other hand, the effectiveness of the coating, as seen previously, seems to be influenced mainly by the thickness and uniformity of the outer layer.

Therefore, the reduction of urea solubility through the coating process allows different release rates, which can contribute to the synchronization of dietary nutrients used by the ruminal microbiota. A better synchronization can collaborate with a greater metabolic activity of ruminal microorganisms, to verify the fact an analysis of the cumulative production of gases was carried out.

### Cumulative gas production

The cumulative gas production technique can be used to verify the behavior of some feeds in the rumen. One of the advantages of this technique is that the final product measured (the gas produced) results directly from the metabolism of microorganisms. Another advantage is that monitoring can be done in short time intervals resulting in greater accuracy of results^14^.

Monitoring of this analysis was performed every five minutes for a total analysis period of 1440 min (24 h). A greater production of gas related to the product F17 can be observed (Fig. 4), followed by the UE, F16 and finally by UP with a lesser amount of gas produced. The efficiency of SRU based products can be variable, as it was possible to observe through this analysis. One of the factors that can influence this effect are the different solubilization rates, previously presented. Another factor is the synchronization between the degradation of carbohydrates and the release of NPN. This synchronization is desirable to obtain a better efficiency in the metabolism of ruminal microorganisms^1^.

Therefore, N sources can be better used when associated with energy sources, because, in this situation, synchronization allows greater efficiency in the microbial process of fixing ammonia in the form of glutamate, reducing nitrogen and energy losses. In addition, the supply of soluble carbohydrates can result in a decrease in ruminal pH, increasing the proportion of ammonia in the form of ammonium ion and, consequently, decreasing its absorption by the ruminal epithelium^15^.

In the formulation of the product F17 the source of carbohydrate present is the cassava starch, feed rich in starch. The UE is a product obtained by extruding corn with urea. Corn is also rich in starch, which may explain the similar results between these products. Probably the release rate of N in the product F17 and in the UE together with the release rate of the carbohydrate present in these products was better synchronized, culminating in the results found.

The F16 product has powdered molasses as its energy source. This feed is rich in fructose (simple carbohydrate) which is immediately degraded in the rumen. This rapid solubilization of molasses, even when coated, may have contributed to the poor synchronization, which led to less gas production (less metabolic activity of ruminal microorganisms).

The only carbohydrate source present in the UP product cumulative gas production test was from standard hay (used as a substrate in all tested products). In general, hays contain mainly carbohydrates with slow ruminal degradation. Thus, the UP product, as it does not have a carbohydrate source in its formulation, presented the lowest result in the cumulative gas production test, corroborating the observations described above.

Table 2 shows the comparison between the degradation speeds during the analysis of cumulative gas production. The highest means were respectively F17 (5.42_a_ mL), UE (5.41_a_ mL), F16 (4.77_b_ mL) and UP (4.75_b_ mL). This analysis allowed evaluating the influence of carbohydrate sources on the metabolism of the ruminal microbiota. The slow release caused by coating with wax along with the time required for the degradation of cassava starch showed a better synchronization with the release of nitrogen supplied by urea, which increased gas production and consequently the efficiency of the supplement.

### Thermogravimetric analysis

In Fig. 5, it is possible to observe the thermogravimetric graphs of the product F16 and its components (Fig. 5a) and of the product F17 and its components (Fig. 5b). It is also possible to view the curves of the first derivative of product F16 and its components (Fig. 5c) and F17 and its components (Fig. 5d). During its decomposition, urea undergoes three endothermic reactions. The first decomposition reaction is the main one where the greatest loss of mass occurs, between 70 and 80% of loss in relation to the initial weight^16^.

Carnauba wax is a material that has been used for coating and encapsulating food because it is a natural material, accepted in food formulations and has a high melting point (82 °C). In the thermogravimetric tests, it is possible to observe that the main loss of mass of this ingredient starts close to 250 °C^17^.

Sugar cane molasses is feed rich in sugar and rapidly fermented in ruminal ambient. The thermal analysis of molasses shows that its temperature of onset of degradation is around 206°C^18^. Cassava starch is a starchy feed, reaching up to 90% of dry matter. The thermogravimetric degradation curve of this feed is characterized by a large peak around 330 °C followed by a second peak close to 490 °C^19^. The results of the thermal analysis observed in the present study and shown in Fig. 5 corroborate those found in the aforementioned literature.

When analyzing the behavior of the curves of products F16 and F17, a similarity with the curve of its pure components can be observed. For example, in Fig. 5d it can be seen that the product F17 has two large peaks related to mass loss. The first degradation reaction shown may be the result of the degradation of urea and carnauba, while the second peak can be related to the loss of mass of the cassava starch and the second reaction of urea degradation.

This similar behavior in the thermogravimetric curve between the products developed in the present work and their components shows that there was no chemical interaction between the components. Despite the reduced solubility conferred by the wax, the components still have their chemical properties intact, which is interesting since chemical interactions could affect the efficiency of these feeds and even the formulation of a diet. The reduction in the amplitude of the curves can be related to the protection provided by the wax against the thermal degradation during the analysis.

Finally, through thermogravimetric analysis, it was possible to verify the maintenance of the chemical characteristics of the ingredients used in the developed supplements, thus guaranteeing the integrity of the nutrients that make up the product formula.

## Conclusion

It is possible to reduce the solubility of urea using carnauba wax as a coating material. In addition, through the cumulative production of gas, it was possible to verify a higher metabolism of the ruminal microbiota by F17 formula (cassava starch associated with urea) which was correlated with a better synchronization caused by the controlled release of its components. Finally, by thermogravimetric analysis, it was possible to verify the integrity of the ingredients after the coating process.

Controlled-release urea is already a product used in ruminant feed. However, as seen in this study, there are coating methods that allow a better use of this type of supplement by the ruminal microbiota. Future research can use coating methods to develop products that improve the efficiency of its ingredients, which can benefit those who work with animal production and seek better production rates.

## Methods

The present study, as well as the urea-based supplements, was developed at the Applied Animal Nutrition Laboratory of the Federal University of Mato Grosso do Sul in Campo Grande, Brazil. All experimental procedures were approved by the Animal Ethics Committee on Animal Use at the Federal University of Mato Grosso do Sul (UFMS) in agreement with the animal welfare guidelines (approval number: 654/2015). All procedures were performed in accordance with the approved protocols, relevant guidelines, and regulations. The study was carried out in compliance with the Animal Research: Reporting of In Vivo Experiments (ARRIVE) guidelines.

### Preparation of the supplement and chemical composition

Two supplements have been developed (F16 and F17). The estimated protein equivalent for each product developed is 150% of crude protein. For this, the ingredients used were urea, sulfur flower, carnauba wax, calcium carbonate, and sugarcane molasses (F16) or cassava starch (F17), all of these purchased from local nutrition companies (Campo Grande, MS, Brazil).

Firstly, pure urea was milled (1 mm sieves), seeking better uniformity in the size of the particles to be covered. Then, in a glass container, milled urea, molasses powder (F16) or cassava starch (F17) and the sulfur flower were homogenized. Separately the carnauba wax was placed in a water bath at 80 °C for melting. During 1 min, the mixture containing urea and the carbohydrate source was also heated in a water bath. Then the wax was slowly poured into the mixture, under constant stirring. After the wax was homogenized with the other ingredients, the mixture was removed from the bath, maintaining the stirring. Finally, the calcium carbonate was sprinkled to the mixture during cooling to room temperature.

Samples of the developed products (F16 and F17) as well the commercial products (UE and UP) were placed in Petri dish and dried in a forced-air oven at 55 °C for 96 h before chemical analysis. Concentrations of DM, MM, CP, and EE were determined according to AOAC^20^. OM was calculated as 100-MM. NDF was determined according to the methodology described by Mertens^21^.

### Microscopy

With a stereoscopic microscope (Cobra Micro Zoom MZ1000, Micros, Hunnenbrunn, Austria), using a 40 × magnification, photos were taken of the developed supplements. A control group, without adding a sulfur flower, was also analyzed for comparison of results.

### Solubilization test

For the solubilization test, the methodology for determining ammoniac nitrogen proposed by Bolsen et al.^22^ was used, with modifications. Rumen fluid was obtained before feeding from four-ruminally fistulated Nellore Steers fed 10 kg of corn silage supplemented with 2.0 kg of concentrate supplement (soybean meal, corn ground, and mineral mix) daily. Initially 2 (± 0.005) g of the developed products (F16 and F17) and of the commercial products UP (polymer-coated urea) and UE (extruded urea), were placed in 250 mL flasks. The predetermined analysis times for the quantification of solubilized nitrogen were 5, 10, 20, 40, 60, 120, 180, 240, 300 and 360 min and for each time and product a triplicate was prepared. Subsequently, 100 mL of ruminal fluid, previously collected, filtered and kept at 39 °C, were added to each flask, purged with CO2 and kept in a water bath (39.5 °C) under constant agitation. At predetermined times, 2 mL of each flask was collected and taken to a test tube containing 5 mL of KOH (2 N), and 13 mL of distilled water, these tubes were taken to the nitrogen distiller. For the recovery of the distillate, a 100 mL conical flask was prepared containing 10 mL of boric acid (2%) and the distillation continued until reaching a total volume of 75 mL. Finally, it was titrated with HCl (0.005 N) and spent volume was used by the following equation to determine the concentration of released nitrogen:

1 $${\text{N}}\,({\text{g}}/{\text{mL}}) = \frac{{{\text{HCL}}\,{\text{volume}}\,{\text{spent}} \times {\text{Correction}}\,{\text{factor}}\,{\text{HCL}} \times 0.005 \times 0.014 \times ~100}}{{2\,{\text{mL}}\,\left( {{\text{sample}}} \right)}}.$$

### Thermal analysis

The thermogravimetric analysis (TGA) was performed by a TGA Q-50 from TA instruments^®^. For this, 0.5 g of sample were used and the analysis took place under an atmosphere of synthetic air with a flow of 60 mL per minute up to a temperature of 900 °C.

### Cumulative gas production

The increase in pressure produced inside the flasks was measured using the automatic Gas Production System (ANKOM^®^). The analyzes were done in duplicate, with measurement of gas pressure produced every 5 min, during a total time of 24 h. Initially, 5 g of a *Brachiaria decumbens* hay was weighed in each flask along with 0.5 g of the developed products and of the UP and UE commercials (for comparison). 100 mL of buffer solution, 25 mL of innocuous rumen were also added to this flask and CO_2_ was purged. A control group was also carried out, containing only the standard hay. The data obtained from gas production were measured in psi and transformed to moles of gas using the ideal gas equation. Soon after, the moles were converted into mL of gas produced using Eq. (2).

2 $$Vx=VjPpsi \times 0.068004084$$

where VX = volume of gas at 39 °C, in mL; VJ = flask headspace, in mL; Ppsi = accumulated pressure recorded by the Gas Production System software.

The in vitro gas production data were analyzed according to the model of Schofield et al.^23^ as follows in Eq. (3):

3 $$y=A/\left\{1+{e}^{\left[2+4*B*\left(C-T\right)\right]}\right\}+D/\{1+{e}^{\left[2+4*e*\right]}\}$$

where y = total gas volume at time T (degradation extent); A = gas volume (ml) of the rapidly degradable fraction; B = degradation rate of fraction A (ml/h); C = lag time (h); D = gas volume (ml) of the slowly degradable fraction; E = degradation rate of fraction D (mL/h).

The parameters of the dual-pool logistic model (Shofield et al.^23^) were estimated by the Gauss–Newton method modified by the SAS NLIN procedure (SAS University Edition, Sas Institute Inc. Cary, CA, USA^24^). The maximum number of interactions used was 100 (one hundred). The criterion used to evaluate the model was the coefficient of determination (R2). The model parameters that best described the mean curve of in vitro gas production of the treatments were submitted to an analysis of variance by the PROC GLM procedure, and the means were compared by the Tukey test in the SAS program^24^. A significance level of 5% was adopted in all statistical analyses.

### Ethics declarations

All ruminal fluid donor animal care and handling procedures were ethically standardized and approved by the Ethics Committee on Animal Use at the Federal University of Mato Grosso do Sul (UFMS) (approval no. 654/2015).

## Data Availability

All data generated or analyzed during this study are included in this published article.
